# Development of a core outcome set for oral health services research involving dependent older adults (DECADE): a study protocol

**DOI:** 10.1186/s13063-020-04531-8

**Published:** 2020-07-01

**Authors:** Sinead Watson, Julie McMullan, Paul Brocklehurst, Georgios Tsakos, Richard G. Watt, Rebecca R. Wassall, Andrea Sherriff, Sheena E. Ramsay, Anup J. Karki, Sayaka Tada, Caroline Lappin, Michael Donaldson, Gerald McKenna

**Affiliations:** 1 Centre for Public Health, School of Medicine, Dentistry and Biomedical Sciences, Queen’s University Belfast, Belfast, UK; 2 NWORTH Clinical Trials Unit, Bangor University, Bangor, UK; 3 Department of Epidemiology and Public Health, University College London, London, UK; 4 School of Dental Sciences, Newcastle University, Newcastle, UK; 5 Glasgow Dental School, University of Glasgow, Glasgow, UK; 6 Institute of Health and Society, Newcastle University, Newcastle, UK; 7 Public Health Wales, Cardiff, UK; 8 Discipline of Endodontics, Operative Dentistry and Prosthodontics, Faculty of Dentistry, National University of Singapore, Singapore, Singapore; 9 Community Dental Service, South Eastern Health and Social Care Trust, Dundonald, UK; 10 Health and Social Care Board, Belfast, UK

**Keywords:** Aged, Frail elderly, Oral health, Consensus, Delphi technique

## Abstract

**Background:**

Oral healthcare service provision for dependent older adults is often poor. For dental services to provide more responsive and equitable care, evidence-based approaches are needed. To facilitate future research, the development and application of a core outcome set would be beneficial. The aim of this study is to develop a core outcome set for oral health services research involving dependent older adults.

**Methods:**

A multi-step process involving consensus methods and including key stakeholders will be undertaken. This will involve identifying potentially relevant outcomes through a systematic review of previous studies examining the effectiveness of strategies to prevent oral disease in dependent older adults, combined with semi-structured interviews with key stakeholders. Stakeholders will include dependent older adults, family members, carers, care-home managers, health professionals, researchers, dental commissioners and policymakers. To condense and prioritise the long list of outcomes generated by the systematic review and semi-structured interviews, a Delphi survey consisting of several rounds with key stakeholders, as mentioned above, will be undertaken. The 9-point Likert scale proposed by the GRADE Working Group will facilitate this consensus process. Following the Delphi survey, a face-to-face consensus meeting with key stakeholders will be conducted where the stakeholders will anonymously vote and decide on what outcomes should be included in the finalised core outcome set.

**Discussion:**

Developing a core set of outcomes that are clinically and patient-centred will help improve the design, conduct and reporting of oral health services research involving dependent older adults, and ultimately strengthen the evidence base for high-quality oral health care for dependent older adults.

**Trial registration:**

The study was registered with the COMET initiative on 9 January 2018 http://www.cometinitiative.org/studies/details/1081?result=true.

## Background

The proportion of older adults aged 65 years and over in the United Kingdom (UK) has been steadily increasing, and it is expected that this trend will continue [1]. This demographic transition towards an ageing society inevitably presents significant challenges for health and social care services. Current healthcare systems are not designed to address the increasing and complex health needs of the ageing population [2].

Dental health services, for instance, have come under immense pressure to deal effectively with the ageing population and to provide high-quality oral health care. Many older adults are now retaining their dentition for longer compared with a few decades ago [3]. Those who are dentate increasingly present with complex restorations (e.g. crowns, bridges, and dental implants) that have a limited lifespan, high levels of oral diseases such as dental decay and periodontal disease, and increases in dry mouth prevalence due to poly-pharmacy [4, 5]. As the population continues to age and the proportion of older adults increases, the number of older adults with oral health problems will increase also.

Poor oral health can have significant impacts on an individual’s general health. For example, reduced dentition can impact dietary intake through the avoidance of important foods, ultimately leading to malnutrition [6, 7]; it can increase the risk of developing respiratory and cardiovascular diseases; and it can impact speech and negatively affect the quality of life [8, 9].

Self-care tends to decline with increasing age; as a result, oral hygiene measures such as tooth brushing can become difficult to maintain, and accessing routine dental services may also be a challenge for some. Many older adults when entering the care system tend to stop receiving routine check-ups [10]. Furthermore, socioeconomic disparities in oral health are apparent in older adults [11], and there is evidence that the increasing costs of care have resulted in inequalities of access to oral health care [12]. Dental diseases can be prevented, but currently, there is often little or no provision being made for the prevention of oral disease in older adults, especially as they become increasingly dependent on care.

A number of systematic reviews have examined the effectiveness of strategies aimed at improving oral health or preventing dental health problems in older adults in long-term care facilities [13–16]. A common issue reported among the reviews was the huge variation in the outcomes and outcome measures used across the included studies, which precludes pooling of data for a meta-analysis. Consequently, this makes it difficult to draw firm conclusions and make informed decisions about what oral health interventions are most effective in this population group.

Furthermore, the majority of oral health research tends to measure and report clinical dental outcomes while not including outcomes that are considered relevant and meaningful to older people [13, 15] and other important stakeholders such as formal and informal carers, family members, clinical experts and healthcare decision makers.

Most of the oral health research involving dependent older adults has focused on those who reside in care homes [13–16]. However, the proportion of older adults who live at home and are cared for by family, friends and formal carers is increasing, and little is known about this population group’s oral health [17]. Accessing routine dental services may be a challenge for some of these people due to mobility constraints, transport difficulties and multiple health conditions. Despite a growing demand for domiciliary oral healthcare provision for this population group, it appears to be on the decline in some areas [10, 18, 19].

To address the issues outlined above, the development and application of a core outcome set (COS) would be essential. A COS represents an agreed set of outcomes that should be measured and reported, as a minimum, in all trials of interventions for a specific condition and other types of research and clinical audits [20, 21]. Core outcome sets can help reduce outcome reporting bias and heterogeneity across studies, which ultimately can facilitate evidence synthesis and prevent research waste. Furthermore, they are developed using consensus methods involving key stakeholders including patients, clinical experts and healthcare decision makers, which ensure the outcomes included are clinically relevant and patient-centred. An organisation known as COMET (Core Outcome Measures in Effectiveness Trials) has been established to help facilitate the development, application and promotion of core outcome sets in various health-related fields [22]. COMET encourages evidence-based COS development and therefore, has developed a handbook which recommends using a structured approach when developing a COS [23].

### Aim

The aim of this study will be to develop a core outcome set (COS) for oral health services research involving dependent older adults.

The objectives of this study are the following:

1. To identify potentially relevant oral health outcomes for dependant older adults in the academic literature and by interviewing key stakeholders
2. To achieve consensus on a COS for oral health services research involving dependent older adults using the Delphi survey technique and face-to-face consensus meeting

### Scope

The COS should be applicable to any type of oral health services research (clinical trials, other types of research and clinical audits) examining the effectiveness of various strategies aimed at improving oral health or preventing dental health problems in dependent older adults. This includes adults who are aged 65 years or over who depend on others to provide some or all their own self-care. This includes people living in care homes (residential and nursing homes) and those who currently live at home.

## Methods/design

**Fig. 1:**
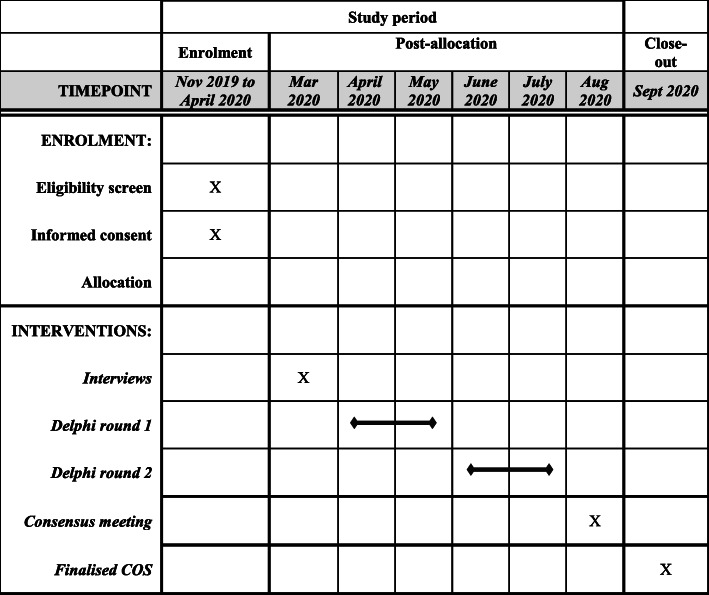
SPIRIT figure; schedule of enrolment and interventions

The development of this protocol was guided by the COMET handbook [23] and is reported in accordance with the Standard Reporting Items: Recommendations for Interventional Trials (SPIRIT) guidelines (Additional file 1) and the Core Outcomes Set-STAndardised Protocol Items (COS-STAP) (Additional file 2). The study’s timeline is shown in Fig. 1.

**Fig. 2:**
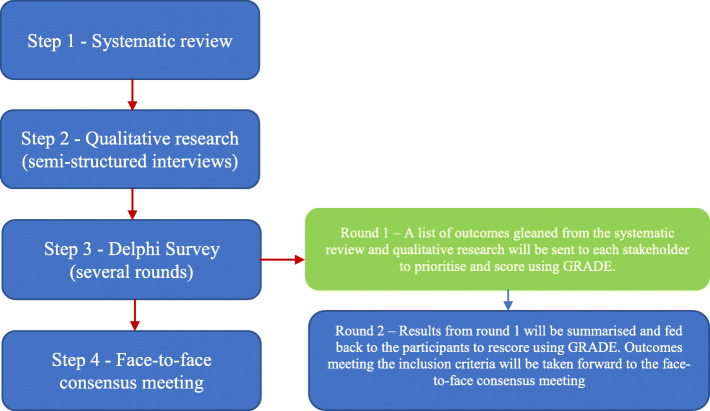
Multi-step process used to develop the COS for oral health services research involving older adults

To develop a core outcome set (COS) for oral health services research involving dependent older adults, a multi-step process (Fig. 2) involving consensus methods with major stakeholders will be undertaken.

### Step 1—Identification of potentially relevant outcomes from academic literature

A systematic review of studies examining the effectiveness of strategies to prevent oral disease in dependent older adults is currently being undertaken to identify an initial list of potentially relevant outcomes. A protocol describing the process of the systematic review has been registered on the PROSPERO database [24].

Briefly, a search will be undertaken of online databases including MEDLINE, CINAHL, Web of Science and EMBASE; trial registries including the International Clinical Trials Registry Platform and ClinicalTrials.gov; the grey literature; and reference lists of included studies. Search terms related to oral health and dependent older adults will be used. Intervention studies that focus on the prevention of dental problems or the improvement of oral health in older dependent adults will be considered. Types of interventions will be preventative or curative treatments as well as educational or behavioural change programmes. The following study designs will be included: randomised controlled trials (RCTs), cluster RCTs, crossover RCTs, non-RCTs and pre-post test studies. Participants should be aged 65 years or over and depend on others to provide some or all of their own self-care. This includes people with no current disease, those with existing disease, those residing in care homes (nursing and residential), or those who reside at home. Studies of participants who are independent enough to attend primary dental care services or have participants who are hospitalised or are edentate will be excluded.

Two review authors will independently assess the titles, abstracts and full texts using the eligibility criteria mentioned above. Following discussion, an agreement will be reached as to which studies will be included. Disagreements between the two reviewers after discussion will be resolved by a discussion with a third reviewer. The type of intervention, duration of follow-up, outcomes and outcome measures will be extracted verbatim from each article included in the review. An initial list of outcomes will be created.

Two review authors will independently assess the certainty of the evidence (high, moderate, low and very low) using the Grades of Recommendation, Assessment, Development and Evaluation (GRADE) system [25]. The GRADEpro software [26] will be used to construct the tables. The same two review authors will also assess the risk of bias for included randomised studies using the criteria outlined in the Cochrane Handbook for Systematic Reviews of Interventions [27] and the ROBINS-I tool (Risk of Bias In non-randomised Studies of Interventions) [28] for included non-randomised studies. Any disagreements between the two reviewers after discussion will be resolved by a discussion with a third reviewer.

### Step 2—Identification of additional outcomes: involvement from key stakeholders

Qualitative data collection methods can help identify what outcomes are relevant to different types of stakeholder groups [29]. Semi-structured interviews with key stakeholders will therefore be undertaken with the aim of identifying the outcomes they consider important and to prioritise the outcomes generated by the systematic review.

#### Participants and recruitment

A total of 30 stakeholders (Table 1) will be purposively recruited from private care homes, from community groups across Northern Ireland and via UK-wide professional bodies (including the British Dental Association, British Dietetics Association and British Geriatrics Society). Links that have already been established through collaborative working and previous research projects conducted by the research team will also be exploited.

**Table 1:** Major stakeholders involved in COS development

	Stakeholder group	Example	Recruitment location
Group 1	Care provider or receiver	• Dependent older adults (residing in a care home and community-dwelling) • Carers (community or care home) • Care-home managers • Family members	• Private care homes across Northern Ireland • Community, retirement and church groups across Northern Ireland
Group 2	Healthcare professional	• Dentists (including community) • Consultants in Dental Public Health • Restorative dentists • Geriatricians • Dieticians	• UK-wide professional bodies (e.g. British Dental Association, British Dietetics Association, British Geriatrics Society)
Group 3	Researcher and expert	• Researchers specialised in ageing • Researchers specialised in oral health • Dental commissioners • Policymakers	• Links that have already been established through collaborative working and previous research projects conducted by the research team

It is anticipated that a sample size of 30 will be sufficient; however, if saturation of ideas and opinions is not reached with this number, further interviews will be conducted as necessary. Every effort will be made to recruit a diverse sample, ensuring all the major stakeholder groups are equally represented.

Older adults (Table 1) will be eligible to take part in the interviews if they are aged 65 years or over and depend on others to provide some or all their own self-care. This includes people with no current disease and those with existing disease, residing in care homes (residential and nursing homes) or those who reside at home. To assess whether the older adults meet the definition of dependent, they will be asked to complete the Barthel Index of Activities of Daily Living [30]. Scores range from 0 to 20, with lower scores indicating increased disability. The same older adults will also be asked to complete the Mini-Mental State Examination (MMSE) questionnaire [31] to assess cognitive function. Those who score over 20 (normal cognition and mild cognitive impairment) and can provide fully informed consent will be included in the study.

#### Data collection and analysis

All participants will be asked to take part in a semi-structured interview lasting between 30 and 60 min, either by telephone or face-to-face at a suitable location. The interviews will be conducted in accordance with a protocol consisting of semi-structured open-ended questions. Topics important for COS development and relevant to each stakeholder group will be addressed, including (1) older adults’ experiences of living with poor oral health/oral disease, (2) views and perceptions of current dental care/services for dependent older adults, (3) outcomes that should be measured (and how) in oral health services research and (4) prioritisation of the list of outcomes generated by the systematic review.

A researcher trained in qualitative data collection methods will undertake all interviews. Interviews will be audio-recorded and transcribed verbatim. NVivo (version 12), a qualitative analysis software, will be used to assist in the management and analysis of transcripts. A thematic analysis, as outlined by Braun and Clarke [32], will be undertaken. This will involve generating a list of key codes, which will lead to the development of a coding scheme. This coding scheme will be applied to all transcripts. Codes will then be grouped into categories leading to key themes being constructed. Outcomes will then be identified from each of the themes. The researcher who conducted the interviews will also analyse the data. A second researcher will undertake a 10% verification check on a subset of transcripts.

The outcomes gleaned from the systematic review and the qualitative interviews will be merged into a long list. All outcomes will be classified into appropriate domains following a research team discussion. To facilitate the process, the use of a suitable outcome domain framework will be considered. All outcomes will be written in lay terms with the medical terminology in brackets. A brief explanation of the outcome will also be provided. An established Patient and Public Involvement (PPI) group, the Belfast Older Person’s PPI Group (BELONG), [33] will be asked to review the list of outcomes to ensure the language and content are appropriate. Improvements will be made following the PPI group’s suggestions.

### Step 3—Consensus building: Delphi survey

To reduce and prioritise the long list of outcomes generated by the systematic review and semi-structured interviews, a Delphi survey involving key stakeholders will be undertaken. The Delphi survey is an iterative consensus method that brings together the opinions of a range of diverse but relevant stakeholders. It ensures anonymity and confidentiality of responses, and it can be circulated to a large number of people in different geographical locations.

#### Participants

Stakeholders, as described above in Table 1, will be purposively sampled. There is no agreement regarding an appropriate panel size for achieving consensus via the Delphi approach [34]. A total of eighty stakeholders will be informed about the study and invited to participate. It is expected with a response rate of ~ 60%, a group of around 50 stakeholders will participate. This sample size should be sufficient to achieve consensus; however, if saturation of data is not reached, further participants will be recruited. Every effort will be made to recruit a heterogeneous sample, with similar numbers of stakeholders recruited from each stakeholder group.

#### Data collection and analysis

The survey will be developed using Survey Monkey [35] and will consist of a long list of combined outcomes, written in lay terms and presented in alphabetical order. If applicable, the medical terms will be in brackets along with a short explanation. Participants will receive an email containing a personal link to the survey. A printable version of the online survey will also be available. All participants will receive detailed instructions (email/letter) on how to complete each round of the survey and the timescale for completion. The email/letter will also emphasise the importance of scoring all the outcomes listed and completing all rounds. For those participants who may require help completing the survey, the researcher will provide support in person if appropriate.

The survey will consist of two rounds initially, but if further prioritisation of outcomes is required, subsequent rounds will be added. Descriptive statistics will be used to analyse the data. Following the completion of a round, responses will be summarised and fed back to the stakeholders producing a refined version. In round 1, participants will be asked to rate each outcome listed using the 9-point Likert scoring system proposed by the GRADE Working Group [36] in which scores of 1 to 3 represent an outcome of limited importance, 4 to 6 important but not critical and 7 to 9 critical. For each outcome, there will also be an ‘unable to score’ category for those participants who feel they may not have the level of expertise to score. To be retained into the second round, an outcome should have 50% or more of the participants scoring it between 7 and 9 and fewer than 15% scoring it as 1 to 3. Equally, the consensus that an outcome is excluded will be defined as 50% or more scoring it as 1 to 3 and fewer than 15% scoring it as 7 to 9.

In round 2, the participants who completed round 1 will be presented with their previous score for each outcome and a mean/median score from each stakeholder group separately. They will then be asked to rescore each of the remaining outcomes. Participants will be advised that they do not have to change their score. Consensus regarding whether an outcome should be included in the list of agreed outcomes taken forward to the face-to-face meeting will be defined as 70% or more of the respondents scoring the measure between 7 and 9 and fewer than 15% scoring it as 1 to 3. Equally, the consensus that an outcome is excluded will be defined as 70% or more scoring it as 1 to 3 and fewer than 15% scoring it as 7 to 9. Outcomes in rounds 1 and 2 that participants have been unable to score or do not meet the above inclusion and exclusion criteria will be taken forward for discussion at the face-to-face consensus meeting.

Participants will have 3 weeks to complete each round (online or printable version). At the start of each week, the researcher will identify those participants who have not completed their round and will send them a reminder (via text message or email).

### Step 4—Consensus building: face-to-face consensus meeting

Following the completion of the Delphi survey, key stakeholders (Table 1) will be asked to take part in a face-to-face meeting held in Belfast. The aims of the meeting are to explore why the outcomes identified by the Delphi survey are considered as important, to address any gaps in the generated list of outcomes and ultimately to confirm the outcomes that will be included in the finalised COS.

A total of 15–20 stakeholders who completed the Delphi survey and are willing to participate will be purposively sampled to take part in the meeting. Every effort will be made to recruit a heterogeneous sample, with similar numbers of stakeholders recruited from each stakeholder group. A researcher will facilitate the meeting, and a PPI representative will chair the meeting to avoid a ‘top-down’ approach, i.e. outcome selection will not just be expert-driven but also patient-centred.

The meeting will include two main activities. The first activity will involve asking the participants to discuss the outcomes from the Delphi survey that lacked agreement, i.e. did not meet either the inclusion or exclusion criteria. There will also be an opportunity at this point to address any gaps in the generated list of outcomes from the Delphi survey. Based on these discussions and the Delphi survey results, the second activity will involve asking the participants to vote anonymously, using GRADE as previously described, what outcomes they feel should be included in the final set. This process will be facilitated using electronic keypads to ensure anonymity.

Consensus regarding whether an outcome should be included in the final list of agreed core outcomes will be defined as 70% or more of the respondents scoring the measure between 7 and 9 and fewer than 15% scoring it as 1 to 3. Equally, the consensus that an outcome is excluded will be defined as 70% or scoring it as 1 to 3 and fewer than 15% scoring it as 7 to 9. If consensus is not reached after two rounds of voting, a majority rules approach will be implemented. Following the above process, the final core outcome set will be agreed and presented in the appropriate domains.

## Publication and dissemination of results

The results of this study will be disseminated via national and international scientific meetings, public health meetings and peer-reviewed journal publications. Implementation activities to promote the uptake and use of the COS developed will include engagement with funders, journal editors, trial registries and regulatory bodies. The finalised COS will be published in the COMET database. A public-friendly summary of the research findings will be produced by the research team with assistance from the BELONG PPI group and will be disseminated to all the stakeholders involved in the study.

## Discussion

This protocol paper describes the multi-step process that will be used to develop a COS for oral health services research involving dependent older adults. It is anticipated the development of a standardised set of outcomes will help improve the design, conduct and reporting of future studies in this research area. It will help reduce outcome reporting bias as the agreed outcomes will be collected and reported as a minimum in future studies. A common issue reported among reviews in this research area is the variation in the outcomes and outcome measures reported across studies [13–16], which precludes pooling of data for a meta-analysis. Developing this COS will also help reduce heterogeneity across future studies, which can enhance the comparability of these studies, ultimately facilitating evidence synthesis in systematic reviews and meta-analyses. Firm conclusions and informed decisions about what oral health interventions are most effective in this population group can then be achieved.

Furthermore, the inclusion of key stakeholders and BELONG throughout the process will ensure the applicability of the intended COS. The outcomes included will be relevant and meaningful to a range of stakeholders including patients, care providers, health professionals and healthcare decision makers.

## Limitations

Some potential limitations of this study are anticipated. Stakeholders that will be recruited for the study will be from a limited number of geographical areas due to practical and resource challenges. For example, organising a face-to-face international meeting can be expensive and challenging for some of the stakeholder groups, specifically the dependent older adults. Potentially, this could impact the generalisability of the COS. Nevertheless, it is expected that there will be many international studies included in the systematic review (step 1), and the Delphi survey (step 3) will be advertised internationally via links that have already been established through collaborated working and previous research projects conducted by the research team.

There is a risk of attrition between Delphi survey rounds, but the research team will try to minimise this by sending a reminder via text message or email at the start of each week. Owing to limited resources, the current study will not determine the most appropriate instruments to measure the outcomes included in the final COS. However, a future study following the procedure recommended by the COSMIN (COnsensus-based Standards for the selection of health Measurement INstruments) initiative [37] will be undertaken at a later stage.

## Conclusion

Developing this COS will ultimately strengthen the evidence base for decision-making regarding the provision of high-quality oral healthcare services fit for ageing populations.

## Project status

The study protocol is version 3 (April 2019). The systematic review and the recruitment of key stakeholders for the semi-structured interviews are currently ongoing. Recruitment began for the step 2 interviews November 2019, and it is expected to continue until August 2020 for the remaining steps of the study.

## Supplementary information

**Additional file 1.** SPIRIT 2013 Checklist: Recommended items to address in a clinical trial protocol and related documents.

**Additional file 2.** COS-STAP checklist.

## Data Availability

Not applicable.

## Abbreviations

BELONG: Belfast Older Person’s Patient and Public Involvement Group
CINAHL: Cumulative Index of Nursing and Allied Health Literature
COMET Initiative: Core Outcome Measures in Effectiveness Clinical Trials
COS: Core outcome set
COSMIN: COnsensus-based Standards for the selection of health Measurement INstruments
EMBASE: Excerpta Medica database
GRADE: Grading of Recommendations Assessment, Development and Evaluation
MMSE: Mini-Mental State Examination
PPI: Patient and Public Involvement
RCT: Randomised controlled trial
UK: United Kingdom
